# Sleep quality of patients with heart failure and associated factors

**DOI:** 10.1590/0034-7167-2024-0244

**Published:** 2024-12-16

**Authors:** Juliana Pessoa de Souza, Danielly Farias Santos de Lima, Oriana Deyze Correia Paiva Leadebal, Maria Eliane Moreira Freire, Simone Helena dos Santos Oliveira, Vinicius Batista Santos, Mailson Marques de Sousa

**Affiliations:** I Universidade Federal da Paraíba. João Pessoa. Paraíba. Brazil; II Universidade Federal de São Paulo. São Paulo. São Paulo. Brazil

**Keywords:** Sleep, Sleep Quality, Sleep Latency, Heart Failure, Cross-Sectional Studies., Sueño, Calidad del Sueño, Latencia del Sueño, Insuficiencia Cardíaca, Estudios Transversales.

## Abstract

**Objectives:**

to assess sleep quality of patients with heart failure and associated sociodemographic and clinical characteristics.

**Methods:**

a cross-sectional study, developed with 88 patients. Sleep quality was assessed by the Pittsburgh Sleep Quality Index. The data were analyzed using descriptive and inferential statistics.

**Results:**

the mean sleep quality score was 8.59 ± 3.60 points. 83% of participants were classified as poor sleepers. The number of hours of sleep was 5.99 ± 1.48. Family income of up to one minimum wage and functional class were significantly associated with poor sleepers. There was a positive correlation between functional class and poor sleep quality.

**Conclusions:**

a high frequency of poor sleepers was identified. Worse scores were associated with family income and symptomatic functional class. Health interventions are necessary to control sleep quality, especially in relation to health functionality.

## RESUMEN

**Objetivos:**

evaluar la calidad del sueño de pacientes con insuficiencia cardíaca y las características sociodemográficas y clínicas asociadas.

**Métodos:**

estudio transversal, desarrollado con 88 pacientes. La calidad del sueño se evaluó mediante el Pittsburgh Sleep Quality Index. Los datos fueron analizados mediante estadística descriptiva e inferencial.

**Resultados:**

el puntaje promedio de calidad del sueño fue de 8,59 ± 3,60 puntos. El 83% de los participantes fueron clasificados como durmientes malos. El número de horas de sueño fue de 5,99 ± 1,48. El ingreso familiar de hasta un salario mínimo y la clase funcional se asociaron significativamente con las personas que duermen mal. Hubo una correlación positiva entre la clase funcional y la mala calidad del sueño.

**Conclusiones:**

se identificó una alta frecuencia de personas que duermen mal. Las peores puntuaciones se asociaron con el ingreso familiar y la clase funcional sintomática. Las intervenciones sanitarias son necesarias para controlar la calidad del sueño, especialmente en relación con la funcionalidad de la salud.

## INTRODUCTION

Sleep is a basic need for maintaining health, well-being, cognitive performance, physiological processes, regulating emotions, physical development and quality of life^(1)^, and is considered by the American Heart Association (AHA)^(2)^ as a new measure for assessing cardiovascular health. The literature shows that poor sleep quality contributes to the development or worsening of cardiometabolic health, mainly as a risk factor for mortality^(2)^.

In this context, there is heart failure (HF), considered a final outcome for most heart diseases with potential for sleep disturbances, given its chronic and progressive nature. HF is a clinical syndrome that encompasses a variety of signs and symptoms, in which heart structures undergo changes that influence the functionality of the heart chambers and/or the left ventricular ejection fraction (LVEF)^(3)^.

Dyspnea on exertion, orthopnea, paroxysmal nocturnal dyspnea, fatigue, tiredness, edema, and exercise intolerance are common clinical manifestations of this chronic condition. These symptoms cause interruption and interfere with sleep continuity, which can lead to insomnia and, more seriously, apnea^(4)^. Therefore, sleep disturbances in patients with HF cause increased secretion of catecholamines, elevated blood pressure, increased oxygen consumption required to meet metabolic needs, and, finally, increased cardiac overload, accentuating unfavorable clinical outcomes in disease management^(5)^.

A systematic review indicates that poor sleep quality in patients with HF has been associated with mood disorders, cognitive disorders, inefficiency, motor impairment, nonspecific physical weakness, and poor quality of life. According to the results of studies, poor sleepers may have difficulty performing self-care behaviors and adhering to treatment, and, consequently, a higher incidence of worse health outcomes^(6)^.

A study conducted in Spain classified 73% of patients with HF as poor sleepers^(7)^. Another study, conducted in the United States of America, showed that 63% of participants had poor sleep quality, and good sleepers were associated with non-white individuals and categorized in functional classes I and II of the New York Heart Association (NYHA)^(8)^. Furthermore, researchers highlighted side effects of medication use associated with impaired sleep quality^(9)^.

Contemporary research aimed at assessing sleep quality in this population is still scarce in Latin America. In Brazil, the prevalence of HF remains high, with 364,475 hospital admissions and a hospital mortality rate of 12.87% in the *Sistema Único de Saúde* (SUS, Brazilian Health System) in the 2021-2022 biennium^(10)^.

In the Northeast region, no studies were found that addressed sleep quality in patients with HF. Therefore, it is important to understand the problem and, in clinical practice, the sociodemographic and clinical conditions that may influence health indicators, implement specific care plans to screen patients at risk for sleep disturbances, and propose non-pharmacological interventions to improve sleep hygiene and therapeutic management.

Considering the above, the following research question was developed: how is the sleep quality of patients with HF presented and what is the association with sociodemographic and clinical characteristics?

## OBJECTIVES

To assess the sleep quality of patients with HF and associated sociodemographic and clinical characteristics.

## METHODS

### Ethical aspects

This research was conducted in compliance with the ethical and legal principles set forth in Resolution 466/12 of the Brazilian National Health Council, and was approved by the Research Ethics Committee. The research participants formalized their consent by signing the Informed Consent Form (ICF), which was made up of two copies.

### Study design, period and location

This is a cross-sectional study based on the STrengthening the Reporting of OBservational studies in Epidemiology (STROBE) recommendations. The study was developed at the cardiology outpatient clinic of a public university hospital located in the city of João Pessoa, PB, northeastern Brazil, from September 2022 to April 2023. In this outpatient clinic, patients are referred by the municipal health network for specialized consultations.

### Population, sample; inclusion and exclusion criteria

The population considered was 112 patients with HF undergoing regular follow-up at the institution. Sample size calculation was performed using the public domain program OpenEpi version 3.01, considering data from a previous study^(11)^, which identified a prevalence of 68.5% of poor sleepers in patients with HF, a 95% confidence level and a 5% sampling error. A minimum sample of 85 participants was obtained.

Patients with a confirmed diagnosis of HF in their medical records, regardless of the etiology and LVEF, and age ≥ 18 years were included. Patients with a medical diagnosis of anxiety, depression and use of sleep-inducing medications, cognitive deficit recorded in their medical records that made it impossible to understand the study objective and respond to or complete the data collection instruments were excluded.

### Study protocol

Data collection occurred with patients pre-scheduled for consultations, by checking medical records for data corresponding to the inclusion and exclusion criteria. Patients were then invited to participate in the research and signed the ICF.

Training was provided to researchers from the research group regarding the questions contained in the instruments, ensuring data collection standardization. Two instruments were used. The first instrument was used to characterize the sociodemographic and clinical profile of patients with HF, used in a previous study^(12)^, with the following variables: sex; date of birth; age; origin; self-reported skin color; marital status; level of education; current employment status; family income; etiology of HF; functional class according to the NYHA classification (I: absence of symptoms; II: symptoms on exertion; III: symptoms on mild exertion; and IV: symptoms at rest); comorbidities associated with HF; risk factors; LVEF obtained by means of a transthoracic echocardiogram report; and drug therapy in use.

Sleep quality was assessed using the Pittsburgh Sleep Quality Index (PSQI), translated and adapted to Brazilian Portuguese^(13)^. This second instrument seeks to assess sleep quality over the last month and combines quantitative and qualitative information, classifying patients as “good sleepers” or “poor sleepers”. The questionnaire consists of 19 questions directed at patients and five at their partners. However, these last questions are only for clinical information, not interfering with the instrument’s total score, and were not applied in this investigation^(13)^.

The PSQI presents 19 questions, grouped into seven components: (1) subjective sleep quality - assesses sleep quality perceived by a person; (2) sleep latency - measures the time needed to fall asleep after lights out; (3) sleep duration - reports the total amount of sleep obtained during the nighttime sleep episode; (4) habitual sleep efficiency - the ratio of total sleep time to time spent in bed (at least 85% of total time); (5) sleep disturbances - measures how often and why someone woke up after falling asleep; (6) use of sleeping medication - assesses how often a person takes medication(s) to fall asleep or stay asleep; and (7) daytime dysfunction - assesses how often a person has trouble staying awake while driving, eating, or engaging in social activities and how much of a problem it is for the person to maintain enthusiasm for doing the activities, with scores from 0 to 3, which can generate a total score that varies between zero and 21 points, and the higher the score, the worse the quality of sleep. People with scores ≥ 5 indicate poor quality of sleep^(9,13)^.

### Analysis of results, and statistics

Data were organized in a Microsoft Excel^®^ spreadsheet and transferred to Statistical Package for the Social Sciences (SPSS) version 22 (IBM Corp., Armonk, NY, USA), analyzed using descriptive and inferential statistics. Numerical variables were presented as mean and standard deviation, and categorical variables as absolute and relative frequencies. Data normality was assessed using the Kolmogorov-Smirnov test, which showed normal distribution. In bivariate analysis, participant sociodemographic and clinical characteristics were compared between two groups using t-test for independent samples.

Pearson correlation analysis was performed to measure relationships between the total PSQI score and the numerical variables. For the ordinal variable, functional class, Spearman correlation was used. To assess the strength of the correlation coefficients, the following criteria were adopted: ≤ 0.30, weak magnitude; between 0.40 and 0.60, moderate magnitude; and above 0.70, strong magnitude^(14)^. The level of statistical significance defined was ≤ 0.05.

## RESULTS

Of the 88 participants, 50 (56.8%) lived in the city of João Pessoa, PB, with a mean age of 57.16 ± 13.20 years; 46 (52.3%) were male; 42 (47.7%) were self-declared brown, with a mean education of 6.32 ± 4.47 years. In addition, 44 (50.0%) were married/stable union; 44 (50.0%) were retired; and 58 (65.9%) had a family income of one minimum wage. As for clinical variables, 62 (70.5%) had a diagnosis of HF of non-ischemic etiology; 37 (42.0%) were in NYHA functional class II, with a mean LVEF of 40.40 (± 13.86); 67 (31.8%) had hypertension associated with HF; 50 (78.1%) did not perform physical activity; 78 (35.1%) used beta-blockers; 68 (30.6%) used diuretics and a mean of 6.30 (± 2.33) medications in use.

Regarding sleep quality, the total PSQI score had a mean of 8.59 ± 3.60, with a minimum score of 1 and a maximum of 16 points. Most participants were classified as poor sleepers, that is, with PSQI scores ≥ 5 points, corresponding to 73 (83.0%) of those investigated. The mean number of hours of sleep was 5.99 ± 1.48. It was found that component 2 - sleep latency - obtained a score of 1.75 ± 1.10, and was the component with the highest mean among the seven components investigated. Table 1 presents the scores of the PSQI components and the total score.

**Table 1:** Pittsburgh Sleep Quality Index component scores, João Pessoa, Paraíba, Brazil, 2023

Variables	Mean ± SD
C.1 Subjective sleep quality	1.36 ± 1.04
C.2 Sleep latency	1.75 ± 1.10
C.3 Sleep duration	1.14 ± 1.15
C.4 Habitual sleep efficiency	1.66 ± 1.17
C.5 Sleep disturbances	1.52 ± 0.56
C.6 Use of sleeping medication	0.00 ± 0.00
C.7 Daytime dysfunction	1.26 ±1.10
Total PSQI score	8.59 ± 3.60

*C - component; SD - standard deviation; PSQI - Pittsburgh Sleep Quality Index.*

Table 2 presents the difficulties encountered by participants in sleeping in the last 30 days, as assessed by the PSQI. It was observed that the most frequent problems in initiating or maintaining sleep were as follows: needing to wake up to go to the bathroom (68.2%); waking up during the night or early in the morning (64.8%); and falling asleep within 30 minutes (42.0%).

**Table 2:** Frequency of difficulties encountered in sleeping, João Pessoa, Paraíba, Brazil, 2023

Variables n (%)	None in the last month	Less than once/week	1 or 2 times/week	3 times or more/week
Could not fall asleep within 30 minutes	31 (35.2)	3 (3.4)	17 (19.3)	37 (42.0)
Woke up in the middle of the night or early in the morning	10 (11.4)	6 (6.8)	15 (17.0)	57 (64.8)
Had to wake up to go to the bathroom	16 (18.2)	3 (3.4)	9 (10.2)	60 (68.2)
Could not breathe comfortably	59 (67.0)	3 (3.4)	7 (8.0)	19 (21.6)
Coughed or snored loudly	53 (60.2)	6 (6.8)	10 (11.4)	19 (21.6)
Felt too cold	75 (85.2)	1 (1.1)	6 (6.8)	6 (6.8)
Felt too hot	46 (52.3)	10 (11.4)	17 (19.3)	15 (17.0)
Had bad dreams	70 (79.5)	6 (6.8)	6 (6.8)	6 (6.8)
Had pain	40 (45.5)	6 (6.8)	9 (10.2)	33 (37.5)
Other reasons	71 (80.7)	-	2 (2.3)	15 (17.0)

Table 3 presents the mean sleep quality scores and sociodemographic characteristics among the groups investigated. Worse scores were observed in the income variable with up to one minimum wage with a significant difference.

**Table 3:** Association of sleep quality and sociodemographic variables, João Pessoa, Paraíba, Brazil, 2023

Variables	n (%)	Mean ± SD	*p* value
Origin			
João Pessoa	50 (56.8)	8.16 ± 3.59	0.200
Other municipalities	38 (43.2)	9.16 ± 3.58	
Sex			
Male	46 (52.3)	8.07 ± 3.80	0.153
Female	42 (47.7)	9.17 ± 3.31	
Age			
≤ 59 years	46 (52.3)	8.96 ± 3.74	0.322
≥ 60 years	42 (47.7)	8.19 ± 3.43	
Skin color			
White	17 (19.3)	7.88 ± 3.62	0.370
Non-white	71 (80.7)	8.76 ± 3.60	
Education			
< 8 years	50 (56.8)	8.32 ± 3.53	0.422
≥ 8 years	38 (43.2)	8.95 ± 3.70	
Marital status			
With partner	44 (50.0)	9.07 ± 3.57	0.216
Without partner	44 (50.0)	8.11 ± 3.61	
Employment situation			
Economically active	8 (9.1)	9.63 ± 3.62	0.398
Economically inactive	80 (90.9)	8.49 ± 3.60	
Income			
Up to a minimum wage	58 (65.9)	9.31 ± 3.47	0.008
More than a minimum wage	30 (34.1)	7.20 ± 3.49	

*SD - standard deviation; NYHA - New York Heart Association; LVEF - left ventricular ejection fraction; HF - heart failure.*

Table 4 presents the mean scores between sleep quality and clinical characteristics. It is noteworthy that the NYHA symptomatic functional class variable presented worse scores with a significant difference.

**Table 4:** Association of sleep quality and clinical variables, João Pessoa, Paraíba, Brazil, 2023

Variables	n (%)	Mean ± SD	*p* value
HF etiology			
Ischemic	26 (29.5)	7.81 ± 3.51	0.188
Non-ischemic	62 (70.5)	8.92 ± 3.61	
NYHA functional class			
Class I (asymptomatic)	21 (23.9)	6.43 ± 3.38	0.001
Class II and III (symptomatic)	67 (76.1)	9.27 ± 3.41	
LVEF			
Reduced LVEF	64 (72.7)	8.89 ± 3.57	0.204
Preserved LVEF	24 (27.3)	7.79 ± 3.62	
Comorbidities			
1-2 comorbidities	40 (45.5)	9.03 ± 3.32	0.305
3 or more comorbidities	48 (54.5)	8.23 ± 3.81	
Medications			
Up to 5 medications	35 (39.8)	8.80 ± 3.48	0.661
More than 5 medications	53 (60.2)	8.45 ± 3.70	

*SD - standard deviation; NYHA - New York Heart Association; LVEF - left ventricular ejection fraction.*

Table 5 presents the correlation coefficients and the sociodemographic and clinical variables. A moderate positive correlation was observed between the total PSQI score and the functional class with a significant difference (p < 0.001). Therefore, the higher the NYHA functional class, the worse the sleep quality.

**Table 5:** Correlations between the Pittsburgh Sleep Quality Index score and the variables of interest, João Pessoa, Paraíba, Brazil, 2023

Variables	Correlation coefficient	*p* value
Age^*^	-1.33	0.214
Education^*^	0.80	0.459
Functional class^**^	0.44	0.001
Number of comorbidities^*^	-0.00	0.935
LVEF^*^	-0.02	0.798
Number of medications^*^	-0.11	0.301

LVEF - left ventricular ejection fraction;

* Pearson correlation;

** Spearman correlation.

## DISCUSSION

This study aimed to assess the sleep quality of patients with HF and associated factors. According to the results, most patients were categorized as poor sleepers (PSQI > 5 points). Participants studied presented altered hours of sleep, with less than 7 hours per night, when it is recommended that adults have 7 to 9 hours of sleep per night, as inadequate hours of sleep represent a greater risk of cardiovascular events and mortality^(6,15)^. It is also known that impaired sleep quality increases symptoms of fatigue and excessive daytime sleepiness, reducing the capacity for rehabilitation and decision-making for daily activities, compromising well-being^(6,9)^.

Studies in different countries have also identified high scores for sleep quality in people with HF. In Iran, the mean PSQI scores were 8.74 ± 2.83^(5)^ and, in Egypt, the PSQI was 9.7 ± 3.4 and ranged from 8.2 to 11.4 points^(16)^. In turn, the studies^(17,18)^ presented higher values, with means of 12.3 ± 4.2 and 14.27 ± 3.32, respectively, supporting the high frequency of poor sleep quality in this population.

Regarding the number of hours of sleep, a similar result was found in a study conducted with 100 patients, in which it was found that 49.5% of those investigated slept between 6 and 7 hours^(5)^. Another study conducted in São Paulo, which assessed sleep quality of patients with acute coronary syndrome (ACS), showed a median of 6 hours of sleep^(19)^. It is known that impaired sleep causes hormonal changes that influence exacerbations of the disease and undesirable hospitalizations, and it is beneficial for heart disease patients to have adequate hours of sleep in order to mitigate cardiovascular events and mortality^(6)^.

Analysis of the PSQI components showed that the sleep latency variable had the highest mean (1.75 ± 1.10), which corresponds to the time required for a decrease in wakefulness and complete sleep onset^(9,13)^. This finding supports a study conducted in Indonesia with 153 patients with HF, which also showed latency as the most affected sleep component^(20)^. Thus, pharmacological interventions have been used to help reduce latency time and increase sleep duration. Sleep hygiene measures, devices to minimize sleep interruptions, educational strategies, phototherapy, relaxation techniques and cognitive therapies show beneficial results and can assist nurses and multidisciplinary teams in promoting sleep quality^(21)^. Therefore, investigating the effect of these actions on the variables that impact sleep quality is timely and may support effective health care planning for people with HF.

Falling asleep in 30 minutes or less, waking up no more than once a night, and staying awake for 20 minutes or less after falling asleep are considered elements of good sleep quality^(1)^. Among the study participants, waking up in the middle of the night or early in the morning and having to wake up to use the bathroom at least three times a week were the main reasons for sleep interruption. One possible explanation may be related to the use of diuretics by participants close to bedtime, favoring sleep interruption due to the need to urinate and/or due to the effects of beta-blockers. In Ethiopian patients with diabetes, hypertension, and HF, similar findings were identified^(1)^. Another investigation conducted in Iran showed a significant relationship between higher PSQI scores and the use of diuretics^(22)^.

Concerning sociodemographic characteristics, there was a predominance of older adults, men, married/stable union and retired, which converges with research carried out in Italy^(9)^. In the present study, sex, age, skin color, marital status and employment status were not associated with sleep quality. In contrast, research carried out in Nigeria showed a significant difference for the sex variable, with worse scores in women compared to men^(23)^. Another study showed that black participants reported worse sleep quality than whites^(24)^. Therefore, prospective longitudinal studies are necessary to establish associations between racial groups and sex with sleep quality.

In this research, higher scores of poor sleepers were identified associated with family income of up to one minimum wage. It is believed that economic status can influence good sleep quality. Access to goods and services favors food consumption, housing conditions and educational level. This argument is supported by research that showed that sleep quality is associated with income satisfaction and, consequently, a better perception of quality of life^(25)^. American research has shown that family income and poverty levels were associated with difficulty initiating sleep and early awakening in patients with HF^(26)^. Therefore, social vulnerability may favor sleep deprivation. However, further studies are needed to confirm this result.

In relation to clinical conditions, NYHA symptomatic functional classes II and III presented the worst scores in sleep quality, i.e., a higher frequency of poor sleepers in patients with mild and/or moderate limitations of symptoms triggered by efforts in carrying out daily activities. Correlation analysis indicated that the higher the NYHA class, the higher the levels of poor sleep quality and PSQI scores. It is worth noting that, since HF is a progressive disease, variation and worsening of symptoms are common. With the advancement of cardiac remodeling, the presence of a poor sleep pattern is likely, with changes in its components and impairment of quality of life^(4)^. This finding is supported by a study carried out in Jordan, which showed a predominance of sleep disturbances in 80% of class II patients and 100% of class III patients^(27)^. Given this result, it is essential that the multidisciplinary team implements interventions, especially with regard to improving functional capacity, since it has been demonstrated that the implementation of cardiopulmonary rehabilitation programs in patients with HF can impact on improving sleep quality and quality of life^(28)^.

In this sample, no significant correlations were found between the number of medications, comorbidities, ejection fraction and sleep quality. However, researchers have identified an association between these variables in hospitalized patients with HF^(29)^. It is known that patients with multiple comorbidities present several pathophysiological, behavioral and psychological changes that cause hospitalizations, poor sleep quality and treatment. The investigation showed that patients with comorbidities associated with HF are three times more likely to have poor sleep quality^(30)^. Thus, the predominance of sleep disturbances in this population is confirmed, and the importance of further research that explores clinical conditions in different health care settings for therapeutic decision-making is highlighted.

This study’s strength is the absence of participants who did not use sleeping medication and were not categorized in NYHA functional class IV, which allows for a closer assessment of perceived sleep quality.

### Study limitations

Data were collected through self-reporting, in a single health center and using a cross-sectional design, which does not allow establishing causal relationships. Therefore, the findings should be assessed with caution. Multicenter, longitudinal designs with larger samples and multivariate analyses are necessary in future research.

### Contributions to nursing, health or public policy

This research presents relevant information on sleep quality, expanding the current state of the art on the subject in a context of northeastern Brazil, and highlights the need for screening for sleep disturbances in patients with HF. Based on factors associated with sleep quality, there is a clear need for patient guidance on non-pharmacological measures to promote sleep and implement cardiopulmonary rehabilitation programs aimed at increasing functional capacity, which can lead to improved sleep quality. Subsequent studies are expected to longitudinally assess the impact of sleep quality on clinical outcomes of patients with HF and the acceptability of sleep hygiene strategies to improve this population’s quality of life.

## CONCLUSIONS

A high frequency of poor sleepers and those with low sleep hours was observed among patients with HF. Sleep latency was the most affected component. It was found that the factors significantly associated with worst sleep quality scores were family income and NYHA functional class. Therefore, non-pharmacological measures are appropriate to promote sleep hygiene, maintain health and, consequently, clinically control sleep quality in patients with HF.
