# ERRATUM

**DOI:** 10.1590/0034-7167.20257801e01

**Published:** 2025-02-03

In the article “Sleep quality of patients with heart failure and associated factors”, with DOI number: https://doi.org/10.1590/0034-7167-2024-0244, published in Revista Brasileira de Enfermagem, 2024;77(6):e20240244, on page 1:

Where it read:

**Submission:** 05-03-2004

Read:

**Submission:** 05-03-2024

