# Supplementary material for: Sleep quality of patients with heart failure and associated factors
Source: Rev Bras Enferm. 2024 Dec 16;77(6):e20240244. doi: 10.1590/0034-7167-2024-0244 (PMC11654507; doi:10.1590/0034-7167-2024-0244)
Supplement: Translation

# Translation: Qualidade do sono de pacientes com insuficiência cardíaca e fatores associados

**DOI:** 10.1590/0034-7167-2024-0244pt

**Authors:** Juliana Pessoa de Souza, Danielly Farias Santos de Lima, Oriana Deyze Correia Paiva Leadebal, Maria Eliane Moreira Freire, Simone Helena dos Santos Oliveira, Vinicius Batista Santos, Mailson Marques de Sousa

**Affiliations:** I Universidade Federal da Paraíba. João Pessoa. Paraíba. Brasil; II Universidade Federal de São Paulo. São Paulo. São Paulo. Brasil

**Keywords:** Sono, Qualidade do Sono, Latência do Sono, Insuficiência Cardíaca, Estudos Transversais.

## Abstract

**Objetivos:**

avaliar a qualidade do sono de pacientes com insuficiência cardíaca e as características sociodemográficas e clínicas associadas.

**Métodos:**

estudo transversal, desenvolvido com 88 pacientes. A qualidade do sono foi avaliada pelo *Pittsburgh Sleep Quality Index*. Os dados foram analisados por meio de estatística descritiva e inferencial.

**Resultados:**

a média de escore da qualidade de sono foi de 8,59 ± 3,60 pontos. 83% dos participantes foram classificados como maus dormidores. A quantidade de horas de sono foi 5,99 ± 1,48. A renda familiar de até um salário mínimo e a classe funcional foram associadas significativamente a maus dormidores. Houve correlação positiva entre classe funcional e má qualidade do sono.

**Conclusões:**

identificou-se alta frequência de maus dormidores. Piores escores foram associados à renda familiar e à classe funcional sintomática. Intervenções em saúde são necessárias para o controle da qualidade do sono, principalmente em relação à funcionalidade em saúde.

## INTRODUÇÃO

O sono é uma necessidade básica para manutenção da saúde, do bem-estar, do desempenho cognitivo, do processo fisiológico, da regulação das emoções, do desenvolvimento físico e da qualidade de vida^(1)^, e é considerado, pela *American Heart Association* (AHA)^(2)^, como uma nova medida para avaliação da saúde cardiovascular. A literatura evidencia que a má qualidade do sono contribui para o desenvolvimento ou a piora da saúde cardiometabólica, principalmente como fator de risco para mortalidade^(2)^.

Nesse contexto, encontra-se a insuficiência cardíaca (IC), considerada um desfecho final para a maioria das doenças cardíacas com potencial para distúrbios de sono, em face da natureza crônica e progressiva. A IC é uma síndrome clínica que abrange uma variedade de sinais e sintomas, na qual estruturas do coração sofrem alterações que influenciam a funcionalidade das câmaras cardíacas e/ou a fração de ejeção do ventrículo esquerdo (FEVE)^(3)^.

Dispneia aos esforços, ortopneia, dispneia paroxística noturna, fadiga, cansaço, edema e intolerância ao exercício físico são manifestações clínicas comuns dessa condição crônica. Esses sintomas causam interrupção e interferem na continuidade do sono, podendo acarretar insônia e, mais gravemente, apneia^(4)^. Logo, os distúrbios do sono em pacientes com IC provocam aumento da secreção de catecolaminas, elevação da pressão arterial, incremento no consumo de oxigênio necessário para atender às necessidades metabólicas e, por último, aumento de sobrecarga cardíaca, acentuando desfechos clínicos desfavoráveis no manejo da doença^(5)^.

Revisão sistemática aponta que a qualidade do sono em pacientes com IC tem sido associada a distúrbios de humor, distúrbios cognitivos, ineficiência, deficiência motora, fraqueza física inespecífica e má qualidade de vida. Segundo resultados dos estudos, maus dormidores podem apresentar dificuldades para realizar comportamento de autocuidado e adesão ao tratamento, e, consequentemente, maior ocorrência de piores desfechos em saúde^(6)^.

Estudo realizado na Espanha classificou 73% dos pacientes com IC como maus dormidores^(7)^. Outra pesquisa, desenvolvida nos Estados Unidos, evidenciou 63% dos participantes com má qualidade do sono, e bons dormidores foram associados a indivíduos de cor não branca e categorizados nas classes funcionais I e II da *New York Heart Association* (NYHA)^(8)^. Ademais, pesquisadores destacaram efeitos colaterais do uso de medicamentos associados à qualidade do sono prejudicada^(9)^.

Investigações contemporâneas direcionadas à avaliação da qualidade do sono nessa população ainda são escassas na América Latina. No Brasil, a prevalência da IC permanece elevada, com registro de 364.475 internações hospitalares e taxa de mortalidade hospitalar de 12,87% no Sistema Único de Saúde (SUS) no biênio 2021-2022^(10)^.

Na região Nordeste, não foram localizados estudos que abordem a qualidade do sono em pacientes com IC. Portanto, é importante conhecer o problema e, na prática clínica, as condições sociodemográficas e clínicas que podem influenciar os indicadores de saúde, a implementação de planos assistenciais específicos no rastreio de pacientes em risco de distúrbios do sono e a proposição de intervenções não farmacológicas para melhorar a higiene do sono e o manejo terapêutico.

Diante do exposto, elaborou-se a seguinte questão de pesquisa: como se apresenta a qualidade do sono de pacientes com IC e qual a associação com características sociodemográficas e clínicas?

## OBJETIVOS

Avaliar a qualidade do sono de pacientes com IC e as características sociodemográficas e clínicas associadas.

## MÉTODOS

### Aspectos éticos

Esta pesquisa foi realizada respeitando os princípios éticos e legais constantes na Resolução n^o^ 466/12 do Conselho Nacional de Saúde, e foi aprovada pelo Comitê de Ética em Pesquisa. Os participantes da pesquisa formalizaram o consentimento por meio da assinatura do Termo de Consentimento Livre e Esclarecido (TCLE), composto por duas vias.

### Desenho, período e local do estudo

Estudo transversal, baseado nas recomendações do *STrengthening the Reporting of OBservational studies in Epidemiology* (STROBE). O estudo foi desenvolvido no ambulatório de cardiologia de um hospital universitário público localizado na cidade de João Pessoa, PB, Nordeste do Brasil, no período de setembro de 2022 a abril de 2023. Nesse ambulatório, os pacientes são referenciados pela rede municipal de saúde para as consultas especializadas.

### População, amostra; critérios de inclusão e exclusão

A população considerada foi de 112 pacientes com IC em *follow-up* regular na instituição. O cálculo de tamanho amostral foi realizado por meio do programa de domínio público OpenEpi, versão 3.01, considerando dados de estudo prévio^(11)^, que identificou prevalência de 68,5% de maus dormidores em pacientes com IC, nível de confiança de 95% e erro amostral de 5%. Obteve-se uma amostra mínima de 85 participantes.

Os critérios de inclusão foram pacientes com diagnóstico de IC confirmado em prontuário, independentemente da etiologia e FEVE, e idade ≥ 18 anos. Os critérios de exclusão foram pacientes que apresentem diagnóstico médico de ansiedade, depressão e em uso de medicamentos indutores do sono, déficit cognitivo registrado em prontuários que impossibilitasse compreender o objetivo do estudo e responder ou preencher os instrumentos de coleta de dados.

### Protocolo do estudo

A coleta de dados ocorreu com os pacientes pré-agendados para as consultas, através da conferência nos prontuários dos dados correspondentes aos critérios de inclusão e exclusão. Na sequência, os pacientes foram convidados a participar da pesquisa, e assinaram o TCLE.

Realizou-se um treinamento com pesquisadores do grupo de pesquisa quanto às perguntas contidas nos instrumentos, garantindo a padronização da coleta de dados. Utilizaram-se dois instrumentos. O primeiro instrumento foi utilizado para caracterização do perfil sociodemográfico e clínico de pacientes com IC, utilizado em estudo prévio^(12)^, com as seguintes variáveis: sexo; data de nascimento; idade; procedência; cor da pele autodeclarada; estado civil; nível de escolaridade; situação de trabalho atual; renda familiar; etiologia da IC; classe funcional segundo a classificação da NYHA (I: ausência de sintomas; II: sintomas aos esforços; III: sintomas aos pequenos esforços; e IV: sintomas em repouso); comorbidades associadas à IC; fatores de risco; FEVE obtida por meio de laudo de ecocardiograma transtorácico; e terapêutica medicamentosa em uso.

A qualidade do sono foi avaliada pelo *Pittsburgh Sleep Quality Index* (PSQI), traduzido e adaptado para o português do Brasil^(13)^. Esse segundo instrumento busca avaliar a qualidade do sono no último mês e combina informações quantitativas e qualitativas, avaliando os pacientes em “bons dormidores” ou “maus dormidores”. O questionário é composto por 19 questões direcionadas ao paciente e cinco aos companheiros, porém essas últimas questões são apenas para conhecimento clínico, não interferindo no somatório do instrumento, e não foram aplicadas nesta investigação^(13)^.

O PSQI apresenta 19 questões, reunidas em sete componentes: (1) qualidade subjetiva do sono - avalia a qualidade do sono percebida pela pessoa; (2) latência do sono - mede o tempo necessário para adormecer após o apagar das luzes; (3) duração do sono - relata a quantidade total de sono obtida durante o episódio de sono noturno; (4) eficiência habitual do sono - relação entre o tempo total de sono e o tempo na cama (pelo menos 85% do tempo total); (5) transtornos do sono - mede quantas vezes e porque alguém acordou após o início do sono; (6) uso de medicamentos para dormir - avalia com que frequência a pessoa toma o(s) medicamento(s) para adormecer ou permanecer dormindo; e (7) disfunção diurna - avalia a frequência com que a pessoa tem problemas para ficar acordada enquanto dirige, come ou se envolve em atividades sociais e o quanto é um problema para a pessoa manter o entusiasmo para fazer as atividades, com pontuações de 0 a 3, podendo gerar escore total que varia entre zero e 21 pontos e, quanto maior for a pontuação, pior é a qualidade do sono. Pessoas com pontuações ≥ 5 indicam má qualidade do sono^(9,13)^.

### Análise dos resultados e estatística

Os dados foram organizados em planilha *Microsoft Excel* ^®^ e transportados para o programa *Statistical Package for the Social Sciences* (SPSS), versão 22 (IBM Corp., Armonk, NY, EUA), analisados por meio de estatística descritiva e inferencial. As variáveis numéricas foram apresentadas por média e desvio padrão, e as variáveis categóricas, por frequências absolutas e relativas. A normalidade dos dados foi avaliada pelo teste de Kolmogorov-Smirnov, que apresentou distribuição normal. Na análise bivariada, as características sociodemográficas e clínicas dos participantes foram comparadas entre dois grupos, usando o teste t para amostras independentes.

A análise de correlação de Pearson foi realizada para medir relações entre o escore total do PSQI e as variáveis numéricas. Para a variável ordinal, classe funcional, empregou-se a correlação de Spearman. Para avaliar a força dos coeficientes de correlações, adotou-se o seguinte critério: ≤ 0,30, fraca magnitude; entre 0,40 e 0,60, moderada magnitude; e acima de 0,70, forte magnitude^(14)^. O nível de significância estatística definido foi ≤ 0,05.

## RESULTADOS

Dos 88 participantes, 50 (56,8%) residiam na cidade de João Pessoa, PB, com a média de idade de 57,16 ± 13,20 anos; 46 (52,3%) eram do sexo masculino; 42 (47,7%) tinham cor parda autodeclarada, com escolaridade média de 6,32 ± 4,47 anos. Além disso, 44 (50,0%) eram casados/união estável; 44 (50,0%) eram aposentados; e 58 (65,9%) tinham renda familiar de um salário mínimo. Quanto às variáveis clínicas, 62 (70,5%) apresentavam diagnóstico de IC de etiologia não isquêmica; 37 (42,0%) estavam em classe funcional II da NYHA, com média da FEVE de 40,40 (± 13,86); 67 (31,8%) tinham hipertensão arterial associada à IC; 50 (78,1%) não realizavam atividade física; 78 (35,1%) utilizavam betabloqueadores; 68 (30,6%) utilizavam diuréticos e média de 6,30 (± 2,33) medicamentos em uso.

Com relação à qualidade do sono, o escore total do PSQI obteve média de 8,59 ± 3,60, com pontuação mínima de 1 e máxima de 16 pontos. A maioria dos participantes foi classificada como maus dormidores, ou seja, com pontuações PSQI ≥ 5 pontos, correspondendo a 73 (83,0%) dos investigados. A média da quantidade de horas de sono foi 5,99 ± 1,48. Verificou-se que o componente 2 - latência do sono - obteve escore de 1,75 ± 1,10, e foi o componente com maior média entre os sete componentes investigados. A Tabela 1 apresenta os escores dos componentes do PSQI e a pontuação total.

**Tabela 1:** Escores dos componentes do *Pittsburgh Sleep Quality Index*, João Pessoa, Paraíba, Brasil, 2023

Variáveis	Média ± DP
C.1 Qualidade subjetiva do sono	1,36 ± 1,04
C.2 Latência do sono	1,75 ± 1,10
C.3 Duração do sono	1,14 ± 1,15
C.4 Eficiência habitual do sono	1,66 ± 1,17
C.5 Transtornos do sono	1,52 ± 0,56
C.6 Uso de medicamentos para dormir	0,00 ± 0,00
C.7 Disfunção diurna	1,26 ±1,10
Escore total PSQI	8,59 ± 3,60

*C - componente; DP - desvio padrão; PSQI - Pittsburgh Sleep Quality Index.*

A Tabela 2 apresenta as dificuldades encontradas pelos participantes para dormir, nos últimos 30 dias, avaliadas pelo PSQI. Observou-se que os problemas mais frequentes para iniciar ou manter o sono foram estes: precisar levantar-se para ir ao banheiro (68,2%); acordar durante a noite ou manhã cedo (64,8%); e adormecer em até 30 minutos (42,0%).

**Tabela 2:** Frequência das dificuldades encontradas para dormir, João Pessoa, Paraíba, Brasil, 2023

Variáveis n (%)	Nenhuma no último mês	Menos 1 vez/semana	1 ou 2 vezes/semana	3 vezes ou mais/semana
Não conseguiu adormecer em até 30 minutos	31 (35,2)	3 (3,4)	17 (19,3)	37 (42,0)
Acordou no meio da noite ou de manhã cedo	10 (11,4)	6 (6,8)	15 (17,0)	57 (64,8)
Precisou levantar para ir ao banheiro	16 (18,2)	3 (3,4)	9 (10,2)	60 (68,2)
Não conseguiu respirar confortavelmente	59 (67,0)	3 (3,4)	7 (8,0)	19 (21,6)
Tossiu ou roncou forte	53 (60,2)	6 (6,8)	10 (11,4)	19 (21,6)
Sentiu muito frio	75 (85,2)	1 (1,1)	6 (6,8)	6 (6,8)
Sentiu muito calor	46 (52,3)	10 (11,4)	17 (19,3)	15 (17,0)
Teve sonhos ruins	70 (79,5)	6 (6,8)	6 (6,8)	6 (6,8)
Teve dor	40 (45,5)	6 (6,8)	9 (10,2)	33 (37,5)
Outras razões	71 (80,7)	-	2 (2,3)	15 (17,0)

A Tabela 3 apresenta os escores médios da qualidade do sono e as características sociodemográficas entre os grupos investigados. Observaram-se piores escores na variável renda com até um salário mínimo com diferença significativa.

**Tabela 3:** Associação da qualidade do sono e variáveis sociodemográficas, João Pessoa, Paraíba, Brasil, 2023

Variáveis	n (%)	Média ± DP	Valor de *p*
Procedência			
João Pessoa	50 (56,8)	8,16 ± 3,59	0,200
Outros municípios	38 (43,2)	9,16 ± 3,58	
Sexo			
Masculino	46 (52,3)	8,07 ± 3,80	0,153
Feminino	42 (47,7)	9,17 ± 3,31	
Idade			
≤ 59 anos	46 (52,3)	8,96 ± 3,74	0,322
≥ 60 anos	42 (47,7)	8,19 ± 3,43	
Cor da pele			
Branco	17 (19,3)	7,88 ± 3,62	0,370
Não branco	71 (80,7)	8,76 ± 3,60	
Escolaridade			
< 8 anos	50 (56,8)	8,32 ± 3,53	0,422
≥ 8 anos	38 (43,2)	8,95 ± 3,70	
Estado civil			
Com companheiro(a)	44 (50,0)	9,07 ± 3,57	0,216
Sem companheiro(a)	44 (50,0)	8,11 ± 3,61	
Situação laboral			
Ativo economicamente	8 (9,1)	9,63 ± 3,62	0,398
Inativo economicamente	80 (90,9)	8,49 ± 3,60	
Renda			
Até um salário mínimo	58 (65,9)	9,31 ± 3,47	0,008
Mais de um salário mínimo	30 (34,1)	7,20 ± 3,49	

*DP - desvio padrão.*

A Tabela 4 apresenta os escores médios entre qualidade do sono e as características clínicas. Destaca-se que a variável classe funcional sintomática da NYHA apresentou piores escores com diferença significativa.

**Tabela 4:** Associação da qualidade do sono e variáveis clínicas, João Pessoa, Paraíba, Brasil, 2023

Variáveis	n (%)	Média ± DP	Valor de *p*
Etiologia IC			
Isquêmica	26 (29,5)	7,81 ± 3,51	0,188
Não isquêmica	62 (70,5)	8,92 ± 3,61	
Classe funcional da NYHA			
Classe I (assintomático)	21 (23,9)	6,43 ± 3,38	0,001
Classe II e III (sintomático)	67 (76,1)	9,27 ± 3,41	
FEVE			
FEVE reduzida	64 (72,7)	8,89 ± 3,57	0,204
FEVE preservada	24 (27,3)	7,79 ± 3,62	
Comorbidades			
1-2 comorbidades	40 (45,5)	9,03 ± 3,32	0,305
3 ou mais comorbidades	48 (54,5)	8,23 ± 3,81	
Medicamentos			
Até 5 medicações	35 (39,8)	8,80 ± 3,48	0,661
Mais de 5 medicações	53 (60,2)	8,45 ± 3,70	

*DP - desvio padrão; NYHA - New York Heart Association; FEVE - fração de ejeção do ventrículo esquerdo; IC - insuficiência cardíaca.*

A Tabela 5 apresenta os coeficientes de correlações e as variáveis sociodemográficas e clínicas. Observou-se correlação positiva de moderada magnitude entre o escore total do PSQI e a classe funcional com diferença significativa (p < 0,001). Logo, quanto maior a classe funcional da NYHA, pior é a qualidade de sono.

**Tabela 5:** Correlações entre o escore do *Pittsburgh Sleep Quality Index* e as variáveis de interesse, João Pessoa, Paraíba, Brasil, 2023

Variáveis	Coeficiente de correlação	Valor de *p*
Idade^*^	-1,33	0,214
Escolaridade^*^	0,80	0,459
Classe funcional^**^	0,44	0,001
Número de comorbidades^*^	-0,00	0,935
FEVE^*^	-0,02	0,798
Número de medicamentos^*^	-0,11	0,301

FEVE - fração de ejeção do ventrículo esquerdo;

* Correlação de Pearson;

** Correlação de Spearman.

## DISCUSSÃO

Este estudo buscou avaliar a qualidade do sono de pacientes com IC e os fatores associados. De acordo com os resultados, a maioria dos pacientes foi categorizada como maus dormidores (PSQI > 5 pontos). Os participantes investigados apresentaram quantidade de horas de sono alterada, com menos de 7 horas por noite, quando o recomendado é que pessoas adultas tenham de 7 a 9 horas de sono por noite, pois inadequadas horas de sono representam maior risco de eventos cardiovasculares e mortalidade^(6,15)^. Sabe-se também que a qualidade do sono prejudicada aumenta os sintomas de fadiga e a sonolência diurna excessiva, reduzindo a capacidade de reabilitação e a tomada de decisão para as atividades diárias, comprometendo o bem-estar^(6,9)^.

Estudos em diferentes países também identificaram escores elevados para a qualidade do sono em pessoas com IC. No Irã, os escores médios do PSQI foram 8,74 ± 2,83^(5)^, e, no Egito, obtiveram-se PSQI de 9,7 ± 3,4 e variação de 8,2 a 11,4 pontos^(16)^. Por sua vez, os estudos^(17,18)^ apresentaram valores mais altos, com médias de 12,3 ± 4,2 e 14,27 ± 3,32, respectivamente, corroborando a elevada frequência de má qualidade do sono nessa população.

Em relação à quantidade de horas de sono, resultado similar foi encontrado em estudo realizado com 100 pacientes, em que se identificou que 49,5% dos investigados dormiram entre 6 e 7 horas^(5)^. Outra pesquisa conduzida em São Paulo, que avaliou a qualidade de sono de pacientes com Síndrome Coronariana Aguda (SCA), evidenciou mediana de 6 horas de sono^(19)^. Sabe-se que o sono prejudicado provoca alterações hormonais que influenciam exacerbações da doença e hospitalizações indesejáveis, sendo benéfico que pacientes cardiopatas cumpram adequadas horas de sono, a fim de atenuar eventos cardiovasculares e mortalidade^(6)^.

A análise dos componentes do PSQI evidenciou que a variável latência do sono apresentou a maior média (1,75 ± 1,10), que corresponde ao tempo necessário para diminuição da vigília e instalação completa do sono^(9,13)^. Esse achado corrobora pesquisa realizada na Indonésia, com 153 pacientes com IC, que também mostrou a latência como o componente do sono mais afetado^(20)^. Nessa direção, intervenções farmacológicas têm sido empregadas para ajudar a reduzir o tempo de latência e aumento da duração do sono. Medidas de higiene do sono, dispositivos para minimizar interrupções do sono, estratégias educacionais, fototerapia, técnicas de relaxamento e terapias cognitivas mostram resultados benéficos e podem auxiliar enfermeiros e equipe multidisciplinar na promoção da qualidade do sono^(21)^. Portanto, investigar o efeito dessas ações nas variáveis que impactam a qualidade do sono é oportuno e poderá subsidiar um planejamento de cuidado eficaz na saúde de pessoas com IC.

Considera-se que adormecer em 30 minutos ou menos, acordar não mais do que uma vez por noite e ficar acordado por 20 minutos ou menos após adormecer são elementos de uma boa qualidade do sono^(1)^. Entre os participantes do estudo, acordar no meio da noite ou de manhã cedo e ter que se levantar para usar o banheiro pelo menos três vezes na semana foram os principais motivos de interrupção do sono. Uma possível explicação pode estar relacionada ao uso de diuréticos pelos participantes próximo ao horário de dormir, favorecendo a interrupção do sono pela necessidade de urinar e/ou devido aos efeitos de betabloqueadores. Em pacientes etíopes com diabetes, hipertensão e IC, achados semelhantes foram identificados^(1)^. Outra investigação realizada no Irã mostrou relação significativa entre maiores escores do PSQI e o uso de diuréticos^(22)^.

Com relação às características sociodemográficas, verificou-se predomínio de adultos mais velhos, homens, casados/união estável e aposentados, que convergem com investigação realizada na Itália^(9)^. No presente estudo, sexo, idade, cor da pele, estado civil e situação laboral não foram associados à qualidade do sono. Em contraponto, pesquisa realizada na Nigéria evidenciou diferença significativa para a variável sexo, com piores escores em mulheres comparado com homens^(23)^. Outra pesquisa mostrou que participantes negros relataram pior qualidade do sono do que brancos^(24)^. Portanto, estudos prospectivos longitudinais são necessários para estabelecer associações entre grupos raciais e sexo com a qualidade do sono.

Nesta pesquisa, identificaram-se maiores escores de maus dormidores associados com renda familiar de até um salário mínimo. Acredita-se que a condição econômica pode influenciar uma boa qualidade de sono. O acesso a bens e serviços favorece o consumo de alimentos, condições de habitação e nível educacional. Esse argumento é sustentado por pesquisa que evidenciou que a qualidade do sono está associada à satisfação de renda e, consequentemente, melhor percepção de qualidade de vida^(25)^. Investigação americana mostrou que a renda familiar e os níveis de pobreza foram associados à dificuldade de iniciar o sono e ao despertar precoce em pacientes com IC^(26)^. Logo, a vulnerabilidade social pode favorecer a privação do sono. Todavia, mais estudos são oportunos para confirmar esse resultado.

No que se refere às condições clínicas, as classes funcionais sintomáticas II e III da NYHA apresentaram os piores escores na qualidade de sono, ou seja, maior frequência de maus dormidores nos pacientes com limitações leves e/ou moderadas de sintomas desencadeados por esforços na realização das atividades diárias. A análise de correlação indicou que, quanto maior a classe da NYHA, maiores os níveis de má qualidade do sono e dos escores de PSQI. Ressalta-se que, sendo a IC uma doença progressiva, logo, a variação e a piora de sintomas são comuns. Com o avanço do remodelamento cardíaco, é provável a presença de padrão do sono ruim, com alteração dos seus componentes e comprometimento da qualidade de vida^(4)^. Esse achado é apoiado por estudo realizado na Jordânia, que evidenciou predomínio de distúrbios do sono em 80% dos pacientes da classe II e 100% da classe III^(27)^. Diante desse resultado, é fundamental que a equipe multidisciplinar implemente intervenções, principalmente no que tange à melhora da capacidade funcional, uma vez que tem sido demonstrado que a implementação de programas de reabilitação cardiopulmonar em pacientes com IC pode impactar a melhora da qualidade do sono e da qualidade de vida^(28)^.

Nessa amostra, não foram encontradas correlações significativas entre número de medicamentos, comorbidades, fração de ejeção e qualidade do sono. No entanto, pesquisadores identificaram associação entre essas variáveis em pacientes hospitalizados com IC^(29)^. Sabe-se que pacientes com múltiplas comorbidades apresentam diversas alterações fisiopatológicas, comportamentais e psicológicas que provocam hospitalizações, má qualidade do sono e tratamento. A investigação mostrou que pacientes com comorbidades associadas à IC têm três vezes mais chance de apresentarem má qualidade do sono^(30)^. Desse modo, ratifica-se o predomínio de distúrbios de sono nessa população, e ressalta-se a importância de novas pesquisas que explorem as condições clínicas em distintos cenários de assistência à saúde para tomada de decisão terapêutica.

Este estudo apresenta como ponto forte a ausência de participantes que não utilizavam medicamentos para dormir e não estavam categorizados na classe funcional IV da NYHA, o que permite uma avaliação mais próxima da qualidade do sono percebida.

### Limitações do estudo

Os dados foram coletados por meio de autorrelato, em único centro de saúde e delineamento transversal, o que não permite estabelecer relações causais. Assim, os achados devem ser avaliados com cautela. Delineamentos multicêntricos e longitudinais, com amostras maiores, e análises multivariadas são necessários em pesquisas futuras.

### Contribuições para as áreas da enfermagem, saúde ou políticas públicas

Esta pesquisa apresenta informações relevantes sobre a qualidade do sono, expandindo o estado da arte atual sobre o tema em um contexto do Nordeste do Brasil, e destaca a necessidade de rastreiro de distúrbios do sono nos pacientes com IC. Com base nos fatores associados à qualidade do sono, é notória a necessidade de orientação do paciente quanto às medidas não farmacológicas para a promoção do sono e a implementação de programas de reabilitação cardiopulmonar visando ao aumento da capacidade funcional, o que pode acarretar a melhora da qualidade do sono. Espera-se que estudos subsequentes avaliem, de forma longitudinal, o impacto da qualidade do sono nos desfechos clínicos de pacientes com IC e a aceitabilidade de estratégias de higiene do sono para favorecer a qualidade de vida dessa população.

## CONCLUSÕES

Constatou-se alta frequência de maus dormidores e com baixa quantidade de horas de sono entre pacientes com IC. A latência do sono foi o componente mais afetado. Verificou-se que os fatores associados significativamente aos piores escores de qualidade do sono foram a renda familiar e a classe funcional sintomática da NYHA. Portanto, medidas não farmacológicas são oportunas para promoção da higiene do sono, manutenção da saúde e, consequentemente, controle clínico da qualidade do sono em pacientes com IC.
