# Supplementary material for: ERRATUM
Source: Rev Bras Enferm. 2025 Feb 3;78(1):e2025n1e01. doi: 10.1590/0034-7167.20257801e01 (PMC11793165; doi:10.1590/0034-7167.20257801e01)
Supplement: Related texts

# Correction: ERRATA

**DOI:** 10.1590/0034-7167.20257801e01pt

No artigo “Qualidade do sono de pacientes com insuficiência cardíaca e fatores associados”, com número DOI: https://doi.org/10.1590/0034-7167-2024-0244pt, publicado no periódico Revista Brasileira de Enfermagem, 2024;77(6):e20240244, na página 1:

Onde se lia:

**Submissão:** 03-05-2004

Lê-se:

**Submissão:** 03-05-2024
